# Asian Citrus Psyllid RNAi Pathway – RNAi evidence

**DOI:** 10.1038/srep38082

**Published:** 2016-11-30

**Authors:** Clauvis N. T. Taning, Eduardo C. Andrade, Wayne B. Hunter, Olivier Christiaens, Guy Smagghe

**Affiliations:** 1 Department of Crop Protection, Faculty of Bioscience Engineering, Ghent University, B-9000 Ghent, Belgium; 2 EMBRAPA Cassava and Fruits, Rua Embrapa, s/n, Cruz das Almas, Bahia, Cep 44380-000, Brazil; 3 U.S. Department of Agriculture, Agricultural Research Service, 2001 South Rock Road, Fort Pierce, FL 34945, USA

## Abstract

*Diaphorina citri*, known as the Asian citrus psyllid, is an important pest of citrus because it transmits a phloem-limited bacteria strongly implicated in huanglongbing (citrus greening disease). Emerging biotechnologies, such as RNA interference, could provide a new sustainable and environmentally friendly strategy for the management of this pest. In this study, genome and functional analysis were performed to verify whether the RNAi core genes are present in the Asian psyllid genome and if the RNAi machinery could be exploited to develop a management strategy for this pest. Analyses of RNAi-related genes in the Asian citrus psyllid genome showed an absence of sequences encoding R2D2, a dsRNA-binding protein that functions as a cofactor of Dicer-2 in *Drosophila*. Nevertheless, bioassays using an *in Planta* System showed that the Asian citrus psyllid was very sensitive to ingested dsRNA, demonstrating a strong RNAi response. A small dose of dsRNA administered through a citrus flush was enough to trigger the RNAi mechanism, causing significant suppression of the targeted transcript, and increased psyllid mortality. This study provides evidence of a functional RNAi machinery, which could be further exploited to develop RNAi based management strategies for the control of the Asian citrus psyllid.

*Diaphorina citri* Kuwayama (Hemiptera: Liviidae)^1^ known as the Asian citrus psyllid (ACP), is an important vector which transmits a phloem-limited bacteria (*Candidatus* Liberibacter asiaticus) strongly implicated in huanglongbing (HLB; citrus greening disease). As the world’s most serious disease of citrus (Bové, *Candidatus* Liberibacter asiaticus)^2^, HLB causes fruit loss and tree death, threatening the viability and production of citrus worldwide^3^. Since there is no natural resistance to HLB infection in any citrus varieties, management of HLB has focused primarily on the control of ACP^4^. Dependence upon intensive insecticide applications to reduce pathogen spread by ACP feeding has resulted in psyllids developing pesticide resistance^5^. Emerging biotechnologies, such as RNA interference (RNAi), using natural gene-based targeting could provide a new sustainable and environmentally friendly management of ACP, and other insect pests of citrus.

RNAi, the process in which double-stranded RNA (dsRNA) exerts a silencing effect on complementary mRNA, has become a useful research tool in entomology for functional genomics. Characteristics such as easy applications, highly specific targeting, and lack of environmental persistence, make RNAi approaches desirable for crop protection against many insect pests^6^,^7^. The RNAi event comprises two major steps: first, the dsRNA should be taken up by cells and then subsequently processed by the cellular core RNAi machinery, thus triggering the silencing of the target gene. These two steps are regulated by many key genes which play central roles in determining the efficacy of RNAi and can result in significant differences in RNAi efficiency between different insect species. For example, while numerous reports of successful RNAi experiments have been reported in Coleoptera^8^,^9^,^10^, RNAi experiments in Lepidoptera are known to be difficult^11^. Several factors play a role in this observed differential RNAi efficiency. Besides the presence of dsRNA degrading enzymes^12^,^13^ and/or viral suppressors of RNAi^14^,^15^, other causes could be the cellular uptake of dsRNA efficiency^16^. All these factors are also influenced by the biology of each insect which differentially expresses the core components of the RNAi machinery. Therefore, to better evaluate the potential of RNAi as a tool in the control of insect pests such as ACP, there is both the need for adequate genetic information concerning RNAi genes and more insight into the uptake and silencing process of RNAi in the pest insect.

This study was designed to verify whether the RNAi machinery is functional in ACP and whether the sensitivity of ACP to ingested dsRNA could induce a significant RNAi response suitable for building a pest management program. *In silico* genome analysis were performed on the ACP genome to identify core RNAi-related genes known to play key roles in small non coding RNA pathways. This was followed by functional studies based on oral bioassays performed on adult ACP, in order to confirm that the RNAi machinery was functional. In view of potential field application, the ‘*in Planta* System’ (iPS) was used for the feeding bioassays^17^. Five ACP-specific dsRNAs designed to target genes associated with different biological process were screened in these feeding bioassays. Using the iPS, ACP, was shown to be very sensitive to ingested dsRNA, and demonstrated a strong RNAi response. The results of this study provide valuable ground information for future studies on the efficacy of RNAi for the control of ACP, as well as for conducting further genome-wide screens to identify genes that could be used for the development of pest management methods against ACP.

## Results

### RNAi core genes in the Asian Citrus Psyllid

**Figure 1:**
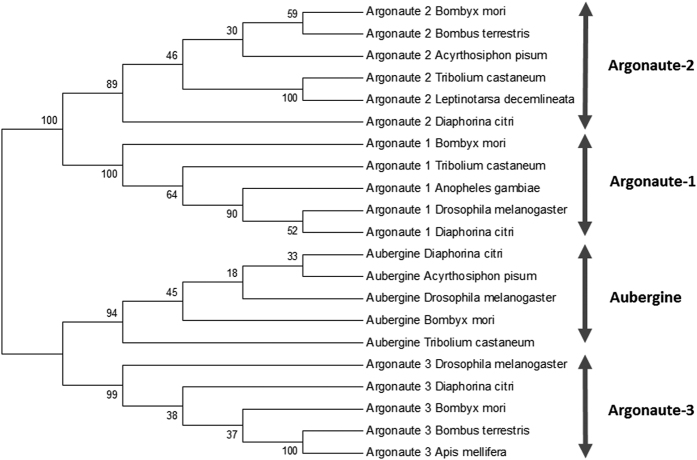
Phylogenetic tree of the Argonaute proteins Ago1, Ago2, Ago3 and Aub. Ago3 and Aub belong to the PIWI-class of Argonaute proteins. Species included in the tree: *Anopheles gambiae* (Diptera); *Apis mellifera* (Hymenoptera); *Acyrthosiphon pisum* (Hemiptera); *Diaphorina citri* (Hemiptera); *Bombyx mori* (Lepidoptera), *Bombus terrestris* (Hymenoptera); *Drosophila melanogaster* (Diptera*); Leptinotarsa decemlineata* (Coleoptera); *Tribolium castaneum* (Coleoptera). The phylogenetic tree was constructed using the Neighbor-Joining method with MEGA, version 6, software. The numbers associated with the branches refer to bootstrap values (confidence limits) resulting from 1000 replicate resamplings.

**Figure 2:**
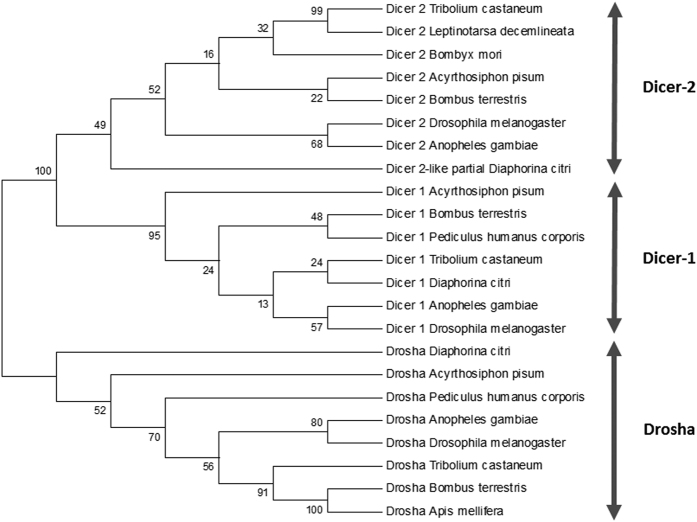
Phylogenetic tree of the Dicer enzymes Dcr1, Dcr2 and Drosha. Species included in the tree: *Anopheles gambiae* (Diptera); *Apis mellifera* (Hymenoptera); *Acyrthosiphon pisum* (Hemiptera); *Diaphorina citri* (Hemiptera); *Bombyx mori* (Lepidoptera), *Bombus terrestris* (Hymenoptera); *Drosophila melanogaster* (Diptera*); Leptinotarsa decemlineata* (Coleoptera); *Pediculus humanus* (Phthiraptera); *Tribolium castaneum* (Coleoptera). The phylogenetic tree was constructed using the Neighbor-Joining method with MEGA, version 6, software. The numbers associated with the branches refer to bootstrap values (confidence limits) resulting from 1000 replicate resamplings.

**Figure 3:**
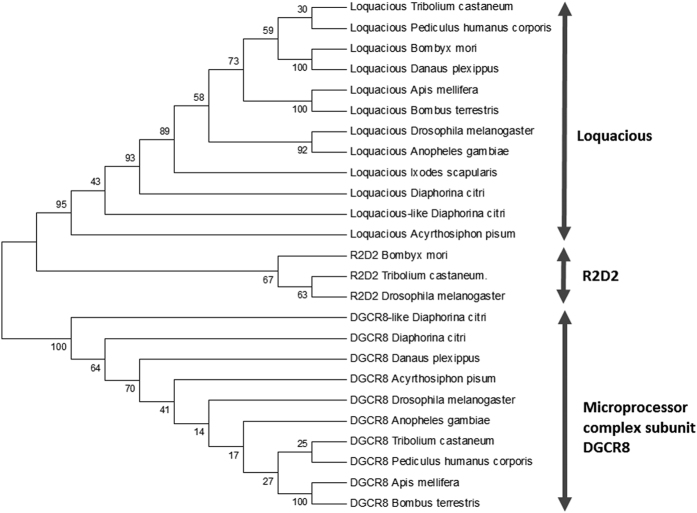
Phylogenetic tree of the Dicer-associated double-stranded RNA-binding proteins Loquacious, R2D2 and Pasha Species included in the tree: *Anopheles gambiae* (Diptera); *Apis mellifera* (Hymenoptera); *Acyrthosiphon pisum* (Hemiptera); *Diaphorina citri* (Hemiptera); *Bombyx mori* (Lepidoptera), *Bombus terrestris* (Hymenoptera); *Danaus plexippus* (Lepidoptera); *Drosophila melanogaster* (Diptera*); Leptinotarsa decemlineata* (Coleoptera); *Ixodes scapularis* (Acari/Ixodidae); *Pediculus humanus* (Phthiraptera); *Tribolium castaneum* (Coleoptera). The phylogenetic tree was constructed using the Neighbor-Joining method with MEGA, version 6, software. The numbers associated with the branches refer to bootstrap values (confidence limits) resulting from 1000 replicate resamplings.

All sequences representing the core RNAi genes were identified in the genome of ACP (Table 1), except R2D2, the cofactor of Dicer-2 in the siRNA pathway. Regarding Dicers in the miRNA pathway, one complete sequence fragment of Dicer-1 (NW_007377676.1), representing the two RNase III domains, was recovered, as well as ~62% of the putative full-length sequence of Drosha (NW_007377741.1). In contrast to Dicer-1, only a partial sequence of Dicer-2 (siRNA pathway, NW_007379804.1) was retrieved, lacking the RNase III domain. With respect to Argonaute proteins, complete or almost complete sequences were identified for Argonaute-1 (miRNA pathway, NW_007377764.1), Argonaute-2 (siRNA pathway, NW_007379505.1) and Aubergine (piRNA pathway, NW_007378348.1), while a smaller fragment for Argonaute-3 (piRNA pathway, NW_007377777.1) was also detected. Finally, complete sequences representing the Microprocessor subunit DGCR8 (NW_007377449.1) and Loquacious (NW_007377608.1) were identified. A phylogenetic tree was constructed for Dicers, Argonaute proteins and dsRNA-binding cofactors that included the ACP sequences and made a comparison with corresponding sequences of organisms of different taxonomic groups (Figs 1, 2 and 3). The phylogenetic trees were meant to confirm the identity of the identified genes from the ACP genome, by comparing the identified RNAi-related genes from the ACP genome with their homologs in other species, hence, providing added confirmation of the identity of these genes.

### Ingestion of ACP specific dsRNA induces RNAi effects and mortality in ACP

Five ACP-specific dsRNAs were designed to target genes associated with different biological processes, namely: (1) Energy mobilization: Arginine Kinase (AK, accession number: GU797832.1)^18^; (2) Cell metabolism: pterin-4-alpha-carbinolamine dehydratase (PCDB1, accession number: DQ673423)^19^; (3) Antioxidant defense: Superoxide Dismutase (SOD, accession number: XM_008474346.1)^20^; (4) Nervous system: Tomosyn, (TOM, accession number: XM_008475017.1)^21^; (5) Reproduction: Vitellogenin (VIT, accession number: XM_008488264.1)^22^.

**Figure 4:**
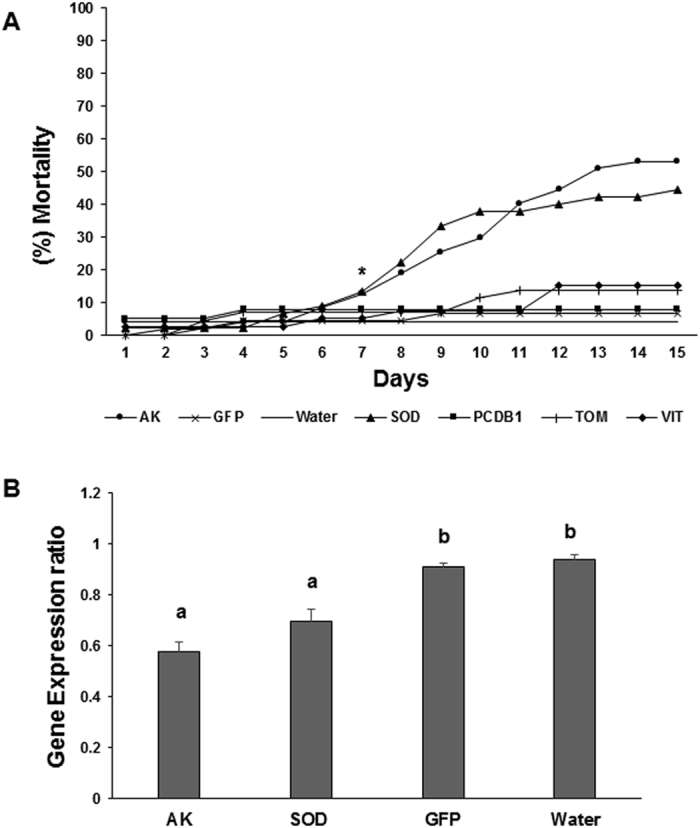
Ingestion of ACP-specific dsRNAs induced insect mortality and gene suppression. (**A**) Adult ACP were fed on citrus flushes treated with dsRNAs targeting five ACP genes and controls treatments (dsGFP and water). ACP mortality was monitored over a 15 days period. The asterisk indicates the first day where cumulative mortality was observed on flushes treated with dsAK or dsSOD, which showed statistical differences (P < 0.05) compared to the controls (dsGFP and water). (**B**) Adult ACP showed reduced levels of AK (Arginine kinase) and SOD (Superoxide Dismutase) mRNAs 5 days post feeding (dpf) on a flush treated with dsAK or dsSOD. Feeding on flushes treated with dsGFP or water did not alter target gene expression in psyllids. Bars represent the standard deviation. Different letters indicate statistically significant differences (P < 0.05).

The dsRNAs were evaluated regarding efficacy to induce RNAi in ACP using the iPS feeding bioassay^17^. ACP were given feeding access for 15 days on citrus flush treated separately with each of the five designed dsRNAs, with a non-ACP dsRNA (dsGFP), and with no dsRNA (water control). ACP overall mortality scored at the end of the 15 days period varied depending on dsRNA ingested (Fig. 4A). Statistically significant mortality was induced only in insects which fed on flushes treated with dsAK (52%) and dsSOD (46%). Non-significant, lower mortality was observed in insects which fed on flushes treated with dsPCDB1 (8%), dsTOM (15%) and dsVIT (16%), which were not statistically different from the control dsGFP (4%) and water (5%) (Fig. 4A). In general, insect mortality started at 6 dpf, increasing overtime reaching a peak at 13 dpf (data not shown). Gene expression analyses were conducted on insects that fed on flush treated with dsAK and dsSOD inorder to confirm that the significant insect death observed was due to an RNAi effect. Insects collected at 5 dpf showed a 58% reduction in mRNA levels for AK mRNA and 69.6% for SOD compared to ACP at starting point (“Time 0”) (Fig. 4B). Insects that fed on flush treated with dsGFP or water did not show significant changes in AK expression levels. Together, these results show that ingestion of dsRNA can trigger the RNAi mechanism in ACP, leading to suppression of the target gene expression causing significant mortality, if the appropriate gene is targeted.

## Discussion

In arthropods it is well established that the core RNAi machinery consists of Argonaute endonucleases, Dicer enzymes and dsRNA binding proteins (Table 1)^23^,^24^,^25^,^26^. Three different RNAi pathways can be distinguished in insects, based on the types of Dicers or Argonautes involved and based on the small RNAs involved. Thus, the siRNA pathway is activated by exogenous dsRNA and involves Dcr2/R2D2 and Ago2 (RNAi core machinery: siRNA pathway, Table 1). The miRNA pathway consists of nuclear Dicer (Drosha/Pasha), cytoplasmic Dicer (Dcr1/Loqs) and Ago1 as core proteins (RNAi core machinery: miRNA pathway; Table 1). The piRNA pathway is also involved in defence against transposable elements and is characterized by Argonaute proteins of the Piwi class (Aub, Ago3) and its independence of Dicer (27 RNAi core machinery: piRNA pathway; Table 1).

Of interest was the finding that the ACP genome lacked sequences encoding R2D2, a dsRNA-binding protein that functions as a cofactor of Dicer-2 in *Drosophila*. In *Drosophila*, *r2d2* is an essential gene for both RNAi and the innate immune response against RNA viruses^28^,^29^. Since RNAi is an efficient process in ACP after dsRNA feeding^17^,^30^,^31^,^32^, it follows that R2D2 may not be necessary for this process in ACP or that it escaped detection. A similar finding was reported by Swevers *et al*.^33^ where *r2d2* was not found in the gut transcriptome of the colorado potato beetle known to have an efficient RNAi process following dsRNA treatment^10^,^34^,^35^. Presumably a different dsRNA-binding cofactor may compensate for the absence of R2D2 in ACP. Interestingly, BLASTX searches using *Tribolium* R2D2 as a query against the ACP genome resulted in the identification of another Loquacious, “Loquacious-like” (NW_007377608.1, Table 1 and Fig. 3). In *Drosophila*, it was demonstrated that Loquacious can act as a cofactor for both Ago1- and Ago2-RISC^36^. Further studies to explore the interaction of Loquacious in the RNAi pathway of ACP may be warranted. With the exception of R2D2, all RNAi-related genes that were searched for in the genome of ACP were identified, often encoding full-length open reading frames (ORFs). This then suggests that ACP has the three functional small RNA pathways, i.e. miRNA, siRNA and piRNA.

Besides these core genes, many different factors were identified that play a role in the efficiency of RNAi (Tables 1 and 2). The idea behind this approach was that RNAi cofactors have co-evolved with the core RNAi machinery and therefore will show similar phylogenetic profiles specific to each arthropod clade^37^,^38^,^39^,^40^,^41^. The picture that emerges is that the different RNAi pathways, while they have distinct components, are intimately integrated with other essential cellular processes such as translation, RNA processing, cytoskeleton function, transcriptional regulation, protein turnover, protein trafficking, splicing, nuclear import and export, DNA repair, and other mRNA degradation pathways^39^,^41^,^42^,^43^,^44^. It was beyond the scope of the present article to investigate the presence of all factors implicated in RNAi in the ACP genome database. However, the identification of the selected RNAi related/associated genes in the ACP genome suggests that these genes do not constitute limiting factors for RNAi efficiency in ACP.

The genomic identification and analysis of core genes for the RNAi pathway, combined with RNAi bioassay results clearly demonstrate that an RNAi response is active in ACP. Similar results from RNAi studies in ACP have also been reported^17^,^30^,^31^,^32^,^45^. However, the results presented here also indicate that, apparently, ACP is very sensitive to RNAi, as a small dose of dsRNA (100 ng) administered through a citrus flush (new growth tissue between 0.25 g to 0.35 g) could induce significant RNAi responses (target gene suppression and death).

Sensitivity in ACP could be due to different factors: (1) an absence or reduced dsRNase activity in the digestive tract and hemolymph; (2) efficient cell uptake of dsRNA, or (3) both. There is no biochemical analysis reported so far showing the presence of nucleases (or degradation of dsRNA) from psyllid salivary enzymes, or in the hemolymph, as shown in aphids^12^. Furthermore, previous studies reported that ingestion of siRNA induced gene silencing and mortality in psyllids^46^. These reports indicate that siRNA can remain intact and functional after being ingested by ACP. Also, these studies show that ACP cells are able to take up siRNA from the environment, and the siRNA could move systemically through the insect body. In Ecdysozoa, two different dsRNA uptake systems have been described so far. On one hand, there are the systemic RNA interference deficient (SID) transmembrane channel-mediated proteins, which were discovered in *Caenorhabditis elegans*^47^. SID-1 homologous genes have been reported from many but not all insects. For the other transmembrane proteins, SID-2 and SID-5^48^, and the tyrosine kinase SID-3^49^, no insect homologs have been reported as of yet. These SID proteins are necessary in the systemic RNAi response in *C. elegans*; for SID-1 and SID-2 it is thought to be through their involvement in dsRNA uptake from the *C. elegans* intestine^47^,^50^. A second mode of uptake of dsRNA in insects is endocytosis. In *Drosophila melanogaster*, no *sid-1* homolog is present, and dsRNA uptake by receptor-mediated endocytosis has been demonstrated for this species^51^,^52^. Furthermore, in the Colorado potato beetle, endocytosis has been shown to be involved in dsRNA uptake, in addition to the SID-1 uptake route^53^. SID-1 was previously shown to occur in two psyllid species, ACP, *Diaphorina citri* and potato psyllid *Bactericera cockerelli L.*^54^, using degenerate primers to the conserved motif of *sid-1*-like genes. These sequences included conserved ‘FYDXHD, MFFSFM, LDDD, and PVF’ amino acid regions. A more thorough examination of SID-1 and other transporters, described from this study provides a better understanding of the genes involved with the RNAi mechanism in psyllids. Analyses of dsRNA movement post oral ingestion from plant tissue in ACP and the glassy-winged sharpshooter, *Homalodisca vitripennis* (Gemar)(Hemiptera: Cicadellidae), reported by Hunter *et al*.^54^ showed dsRNA detection in the fat body post feeding access. However, while SID-1 is a key component in the uptake of dsRNA in some arthropods^55^, the systemic movement in arthropods is not solely dependent upon the presence of SID-1^47^,^56^. It is interesting to note that in two closely related coleopteran insects, *Tribolium castaneum* (Tc) and *Diabrotica virgifera* (Dv), the former has three different *sid-1*-like genes, which do not seem to be necessary for dsRNA uptake^57^ whereas the latter only has two, which are both confirmed as being involved^58^. Further studies will therefore be needed to elucidate the mechanism of dsRNA uptake and systemic spread in ACP. However, the fact that the RNAi machinery is present and can be induced by low amounts of dsRNA delivered through plants and iPS, offers some intriguing possibilities for future applications in pest management.

In conclusion, this study provides evidence of a functional RNAi machinery in the Asian citrus psyllid. Given that ACP has all the important RNAi core genes needed to present a significantly strong response to ingested dsRNA, management strategies based upon RNAi appear to be a suitable approach to successfully reduce this pest in a highly specific manner. Nevertheless, further genome-wide screens to identify better genes, which could induce stronger RNAi effects in ACP will be required for the development of a proper and effective management method for this pest.

## Materials and Methods

### *In silico* identification and manual curation of RNAi-related genes in ACP

A list of RNAi-related genes was composed based on literature (Table 2) that included the following categories: RNAi core machinery, auxiliary factors, dsRNA uptake, antiviral RNAi and nucleases. Amino acid sequences of homologs for these genes were found in GenBank for *Tribolium castaneum*, which has a well-annotated genome. Subsequently, the genome of ACP available on the i5k Workspace@NAL platform (https://i5k.nal.usda.gov/webapp/blast/)^59^, was searched against the genes in this list, using sequences of homologs as query. Hits (contigs) obtained from this search were subsequently used in BLASTX searches to verify their identity and to detect functional domains.

### Phylogenetic analysis of RNAi core genes from ACP genome

To search for ACP’s RNAi homologous proteins in other species, a protein–protein BLAST search (BLASTp) was performed using the NCBI BLAST Service (http://www.ncbi.nlm.nih.gov/). The selected species were: *Tribolium castaneum* (Coleoptera), *Leptinotarsa decemlineata* (Coleoptera), *Bombyx mori* (Lepidoptera), *Danaus plexippus* (Lepidoptera), *Acyrthosiphon pisum* (Hemiptera), *Bombus terrestris* (Hymenoptera), *Apis mellifera* (Hymenoptera), *Pediculus humanus corporis* (Phthiraptera), *Ixodes scapularis* (Acari/Ixodidae), *Drosophila melanogaster* (Diptera) and *Anopheles gambiae* (Diptera). For protein alignment and phylogenetic tree construction, only the hit with the lowest E-value was chosen. If no significant hit was found or the protein sequence obtained was incomplete, the organism was excluded from the phylogenetic tree. Amino acid sequence alignments and phylogenetic analyses were performed with the ClustalW program, integrated in the Molecular Evolutionary Genetics Analysis 6.06 software (MEGA 6.06)^60^. Phylogenetic trees and P-Distances were constructed using the Neighbor-Joining method^61^ with MEGA 6.06 software. Bootstrapping^62^ was used to estimate the reliability of phylogenetic reconstructions (1000 replicates).

### Plant material and insect colony

Citrus plants, cultivar ‘Carrizo’ citrange (*Citrus sinensis* × *Poncirus trifoliata*), used in this study were maintained in a greenhouse under natural light and temperature. The plants were constantly pruned to promote growth of new foliar shoots, referred to as “flush”. The ACP colony was reared on *Citrus macrophylla* in a glasshouse (22 °C) and natural light. Adult ACP, approximately 5 days post eclosion, were used for the experiments.

### Synthesis of double stranded RNA

The synthesis of dsRNAs was performed using the MEGAscript RNAi kit (Thermo-Fisher Scientific) according to the manufacturer’s instructions. The template DNA for dsRNA production was generated by PCR amplification using gene-specific primers containing T7 promoter sequences tailed at the 5′ end of each primer. All primer sequences are listed in Table 2. A dsRNA specific for Green fluorescent protein (dsGFP, AJ306911.1) was used as a negative control in the experiments. After synthesis, dsRNA concentrations were measured using NanoDrop™ ND1000 spectrophotometer (Thermo-Fisher Scientific, Waltham, USA).

### Feeding Bioassay

**Figure 5:**
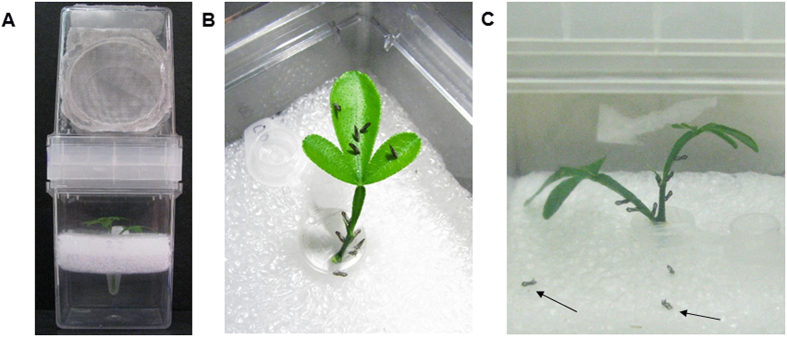
RNA feeding bioassay (*in plant system*, iPS) for dsRNA delivery to *D. citri*. (**A**) Cage containing a citrus flush previously treated with dsRNA solution or water. (**B**) Adult ACP were given feeding access for 15 days. (**C**) Dead insect (indicated with an arrow) can be easily observed at the bottom of the cage few days after feeding.

We used the bioassay called ‘in planta system’ (iPS)^17^ to deliver dsRNA to ACP. Briefly, citrus flushes were collected from potted plants, washed in 0.2% bleach for 10 min and rinsed for 3 min by submersion in autoclaved or filtered water. The base of stems were cut with a razor blade, ~45° angle, while submerged in water. The flush was then transferred to a 1.5 mL tube containing 0.5 mL water. A dsRNA solution (i.e. 100 ng of dsRNA in 300 μL of water) was added to the tube, the tube top was wrapped with Parafilm™ (American National Can™, Neenah, WI 54956) and placed under artificial lighting to stimulate absorption of the dsRNA solution. After the plant flush absorbed the entire dsRNA solution, the tube was filled with filtered water (0.45 μm, Sterile Syringe Filters, Corning^®^). The treated flushes in the tubes were then transferred to a cage and 15 adult ACP, of mixed genders, were added to each cage (Fig. 5). Each treatment (ACP-dsRNA, dsGFP and water control) consisted of four cages, and experiments were repeated three times. Mortality was scored daily for 15 days. Data for total mortality was analyzed with analysis of variance (ANOVA) and t-Test, with P < 0.05 of probability.

### Sample collection, RNA extraction and quantitative real-time PCR (RT-qPCR)

To assess the extent of RNAi by ACP, the level of the mRNA was quantified by RT-qPCR. Each treatment was composed of three cages, with 15 adults in each cage. From each cage, three pools of 3 insects each were collected 5 days post feeding (dpf) on treated flush. At time point zero (the day the experiment was set up) three pools of 3 insects were collected from each treatment group. These samples represent “Time 0” and were used for comparative gene expression analysis. The experiment was repeated twice. ACP were collected using a mouth-operated aspirator (BioQuip) and transferred to 1.5 mL tubes containing 50 μL of Trizol^®^ (Thermo-Fisher Scientific) and glass beads ≤ 106 μm (Sigma-Aldrich). After maceration using a pestle, 950 μL of Trizol^®^ was added to the tube and insects’ total RNA was extracted following the manufacturer’s instructions. An aliquot of 5 μg of total RNA was treated with Turbo DNA-*free*™ (Thermo-Fisher Scientific) to remove residual DNA. The cDNA synthesis was carried out using the SuperScript™ III first-strand synthesis system (Thermo-Fisher Scientific), following the manufacturer’s instructions. RT-qPCR reactions were performed in triplicates using Power SYBR^®^ Green Master Mix (Applied Biosystems, ThermoFisher Scientific). RT-qPCR reactions were carried out using a Rotor-Gene 6000 PCR machine (QIAGEN Corbett, Australia). The α-tubulin expression levels were used to normalize Ct values. The relative gene expression data were analyzed using the relative 2^−ΔΔCT^ method^63^. The expression levels of the target gene were relative to the expression of the same gene in the ACP collected at “Time 0” (which was assigned the value of 1.0). The data were statistically analyzed using analysis of variance (one way ANOVA) and t-Test at P < 0.05 of probability. All the primer sequences used for RT-qPCR are provided on the Table 3.

### Data accessibility

All available genome and transcriptome data are free NCBI-approved version of the genome. For genome data, navigate to the files that we have available here: https://i5k.nal.usda.gov/Diaphorina_citri. No registration is required to download the files. Blast analyses against the *D. citri* DIACI files: https://i5k.nal.usda.gov/webapp/blast/ and browse the genome (no login required) at: https://apollo.nal.usda.gov/diacit/jbrowse/ (NAL: Monica Poelchau, Chris Childers)^65^.

## Additional Information

**How to cite this article**: Taning, C. N. T. *et al*. Asian Citrus Psyllid RNAi Pathway – RNAi evidence. *Sci. Rep.* **6**, 38082; doi: 10.1038/srep38082 (2016).

**Publisher's note:** Springer Nature remains neutral with regard to jurisdictional claims in published maps and institutional affiliations.

## Figures and Tables

**Table 1:** RNAi-related genes identified in the genome of ACP and their Accession IDs.

Group	RNAi-related* gene	Present in the genome	Accession ID	Query^a^ cover	E-value^b^	Identity^c^
RNAi core machinery: siRNA pathway	Dicer-2	Yes	NW_007379804.1	67%	8e-25	38%
	R2D2	No	/	/	/	/
	Ago-2	Yes	NW_007379505.1	99%	2e-86	56%
RNAi core machinery: miRNA pathway	Drosha	Yes	NW_007377741.1	77%	0.0	64%
	DGCR8	Yes	NW_007377449.1	77%	1e-141	46%
	Dicer-1	Yes	NW_007377676.1	79%	0.0	65%
	Loquacious	Yes	NW_007377608.1	96%	2e-71	43%
	Loquacious-like	Yes	NW_007377495.1	84%	1e-73	43%
	Ago-1	Yes	NW_007377764.1	96%	0.0	86%
RNAi core machinery: piRNA pathway	Piwi/Aubergine	Yes	NW_007378348.1	92%	2e-21	38%
	Ago-3	Yes	NW_007377777.1	94%	0.0	41%
Auxiliary factors (RISC)	FXMR	Yes	NW_007377542.1	54%	2e-80	49%
	RNA helicase DDX	Yes	NW_007377487.1	89%	0.0	76%
	Staufen-RA	Yes	NW_007378277.1	18%	5e-41	67%
	Staufen-RB	Yes	NW_007378277.1	63%	6e-17	44%
	Maelstrom	Yes	NW_007378195.1	74%	1e-13	24%
	PRMT5	Yes	NW_007377723.1	99%	1e-44	40%
	Clp-1	Yes	NW_007378998.1	95%	4e-177	58%
DsRNA uptake	Sid-1	Yes	NW_007377471.1	99%	0.0	46%
Antiviral RNAi	Ars2-RA	Yes	NW_007377554.1	78%	5e-151	55%
	Ars2-RB	Yes	NW_007377554.1	42%	5e-30	48%
	Egghead	Yes	NW_007378275.1	85%	7e-149	70%
Nuclease	Nibbler (mut-7)	Yes	NW_007378502.1	55%	2e-23	34%

^*^See Table 2 for cited references for all of the RNAi-related genes used in this study.

^a,b,c^Based on protein-protein BLAST with query sequences from *T. Castaneum* (Coleoptera) against *D. citri* (Hemiptera).

**Table 2:** Overview of RNA interference-related genes investigated in this study, with a brief description of the function.

RNAi core machinery: siRNA pathway		
Dicer-2	RNaseIII, processing of long dsRNA into siRNAs	24–27
R2D2	dsRNA-binding, co-factor of Dicer-2	
Ago-2	Argonaute, catalytic subunit of RISC	
RNAi core machinery: miRNA pathway		
Drosha	RNase III, cleavage of pri-miRNA to pre-miRNA	24–27
DGCR8	dsRNA-binding, co-factor of Drosha	
Dicer-1	RNase III, conversion of pre-miRNA to miRNA	
Loquacious	dsRNA-binding, co-factor of Dicer-1	
Ago-1	Argonaute, catalytic subunit of RISC	
RNAi core machinery: piRNA pathway		
Piwi/Aubergine	Argonaute (PIWI subfamily), catalytic subunit of RISC	28
Ago-3	Argonaute (PIWI subfamily), catalytic subunit of RISC	
Auxiliary factors (RISC)		
FXMR	Fragile-X-related protein, component of RISC complex in S2 cells, RNA binding RGG and KH motifs	66
RNA helicase DDX	DEAD-box RNA helicase, required for RNAi	67
Staufen	Tubulin-binding & dsRNA-binding domain transport of	68
Neuron-specific Staufen	mRNA	
Maelstrom	piRNA pathway domain weak similarity to HMG box, Mael domain with weak homology with the DnaQ-H 3′-to-5′ exonuclease	69
PRMT5	Protein methyltransferase; methylates Piwi proteins at conserved Arg-residues	70
Clp-1	RNA kinase, phosphorylation of siRNAs	71
DsRNA uptake		
Sid-1	Homolog of putative dsRNA transporter in *C. elegans*	72
Antiviral RNAi		
Ars2	Regulator of siRNA- and miRNA-mediated silencing, suppressor of RNA virus infection	73
Egghead	Seven transmembrane- domain glycosyltransferase, uptake of dsRNA, innate immunity against RNA virus (Drosophila)	52
Nuclease		
Nibbler (mut-7)	Processing of 3′ends of miRNAs (Drosophila)	74

**Table 3:** Primers used for dsRNA production and RT-qPCR.

Name	Sequence$	Position*
dsAK-F	TAATACGACTCACTATAGGGAGTGGCATTCTTGTATGGCGTA	57
dsAK-R	TAATACGACTCACTATAGGGAGGCCTGCAAGAATCTGTCTCC	730
dsPCDB1-F	TAATACGACTCACTATAGGGAGGTTCACTGGAGCTAGGAGTGG	3
dsPCDB1-R	TAATACGACTCACTATAGGGAGATTGTATCCATGAATGAGGC	410
dsSOD-F	TAATACGACTCACTATAGGGAGGCGACTCCGGCATTTATC	69
dsSOD-R	TAATACGACTCACTATAGGGAGGAAGTGTTCTTTGTGGAATAGATAGG	847
dsTOM-F	TAATACGACTCACTATAGGGAGTTTCGGTGATTTCAGGAACTG	45
dsTOM-R	TAATACGACTCACTATAGGGAGCGAGGAATTTTTCAAGATGG	446
dsVIT-F	TAATACGACTCACTATAGGGAGAGAACAGATCCCAAGAACCC	2241
dsVIT-R	TAATACGACTCACTATAGGGAGTTGTGATTTTTCAGCTGGGG	2769
dsGFP-F	TAATACGACTCACTATAGGGAGCCAACACTTGTCACTACTTTCTCTT	1
dsGFP-R	TAATACGACTCACTATAGGGAGGTAATGGTTGTCTGGTAAAAGGA	480
AK_quant-F	CGGACTTGAGGGAGAACTGA	611
AK_quant-R	GTGGTAGATACCGCGACCAG	776
α-Tub-F	GCGTCTCTTCGGTTTGACGG	896
α-Tub-R	CACTTCACCATCTGGTTGGC	1092

^$^The underlined sequences correspond to the T7 sequence.

^*^The position of the primer is related to the gene.
